# Embryonic Origin of Olfactory Circuitry in *Drosophila*: Contact and Activity-Mediated Interactions Pattern Connectivity in the Antennal Lobe

**DOI:** 10.1371/journal.pbio.1001400

**Published:** 2012-10-02

**Authors:** Lucia L. Prieto-Godino, Soeren Diegelmann, Michael Bate

**Affiliations:** Department of Zoology, University of Cambridge, Cambridge, United Kingdom; VIB and KU Leuven, Belgium

## Abstract

Olfactory neuropiles across different phyla organize into glomerular structures where afferents from a single olfactory receptor class synapse with uniglomerular projecting interneurons. In adult *Drosophila*, olfactory projection interneurons, partially instructed by the larval olfactory system laid down during embryogenesis, pattern the developing antennal lobe prior to the ingrowth of afferents. In vertebrates it is the afferents that initiate and regulate the development of the first olfactory neuropile. Here we investigate for the first time the embryonic assembly of the *Drosophila* olfactory network. We use dye injection and genetic labelling to show that during embryogenesis, afferent ingrowth pioneers the development of the olfactory lobe. With a combination of laser ablation experiments and electrophysiological recording from living embryos, we show that olfactory lobe development depends sequentially on contact-mediated and activity-dependent interactions and reveal an unpredicted degree of similarity between the olfactory system development of vertebrates and that of the *Drosophila* embryo. Our electrophysiological investigation is also the first systematic study of the onset and developmental maturation of normal patterns of spontaneous activity in olfactory sensory neurons, and we uncover some of the mechanisms regulating its dynamics. We find that as development proceeds, activity patterns change, in a way that favours information transfer, and that this change is in part driven by the expression of olfactory receptors. Our findings show an unexpected similarity between the early development of olfactory networks in *Drosophila* and vertebrates and demonstrate developmental mechanisms that can lead to an improved coding capacity in olfactory neurons.

## Abstract 2

The first study of the embryonic development of the *Drosophila* olfactory network reveals unexpected similarities with vertebrate systems.

## Author Summary

The mechanisms underlying the patterning of connectivity in the insect olfactory system are radically different from those found in vertebrates, but to date most studies in insects have focused on the development of the adult olfactory network. Here, for the first time, we report how larval olfactory circuitry is formed in the embryo of the fruitfly *Drosophila*. By labelling developing sensory neurons and interneurons from the earliest stages to maturity, we find that the patterning of the antennal lobe in *Drosophila*, like the olfactory bulb in mouse, is pioneered by ingrowing sensory afferents, and that interneuronal development depends on the terminals of these pioneering afferents. We also find that antennal lobe patterning depends on contact and activity-mediated interactionsbetween its component cells, as it does in vertebrates. Finally, we report the results of electrophysiological recordings in developing embryos, the first of their kind in any developing olfactory network. We conclude that fundamental mechanisms of circuit assembly and patterning are conserved between *Drosophila* and vertebrates.

## Introduction

The discontinuous glomerular map at the first relay for olfactory information in vertebrates and insects (olfactory bulb and antennal lobe, respectively) is an important model for developmental mechanisms by which neurons assemble into functional neural networks [1]–[6]. This is specially so for the adult olfactory system of *Drosophila*, since identification of its odorant receptor genes (OR) [7]–[9] has made it possible to dissect the organization of the olfactory circuit, and confirms previous ideas of common design principles in vertebrate and insect olfactory systems [10]–[12].

The study of developmental mechanisms that lead to the formation of olfactory circuits in mice and in adult *Drosophila* has shown that different strategies are used in the two organisms. In mice, olfactory sensory neurons (OSNs) lead the process of glomerulus formation and influence the dendritic development of mitral and tufted cells (the projection neurons of the olfactory bulb) [13]–[16]. In marked contrast to this, development of the adult olfactory system in *Drosophila* begins with the positioning of projection neuron dendrites in glomerular-sized territories before the arrival of OSN axons [2]. The adult PNs develop independently of the adult OSNs [17], and the initial positioning of their dendrites depends partly on signals provided by pre-existing larval OSNs [18]. Thus, larval OSNs play a significant role in patterning the adult olfactory network. The role of activity in the development of the two systems is also different. While blocking activity or synaptic transmission in OSNs during development in mice or zebrafish shows that activity is essential for development and refinement of the olfactory map [19],[20], similar experiments have failed to show any such developmental effects in adult *Drosophila* [21]. In none of these systems, however, has the pattern of neuronal activity during development been documented and this means that the results are difficult to interpret because the patterns of activity that are being blocked are unknown.

The larval olfactory system of *Drosophila* shares organizational principles and all the experimental advantages of its adult counterpart, but is numerically much simpler. It consists of 21 OSNs with their cell bodies grouped in an anterior ganglion (Dorsal organ ganglion, DOG). These neurons send dendrites to the dorsal organ (DO), where odour volatiles are detected, and axons into the CNS, where they terminate in the antennal lobe (AL). Each of the OSNs expresses a different OR and sends its axons to a different glomerulus. Thus, unlike the adult or indeed vertebrate systems, in the larva there is no convergence of OSN axons, and every OSN constitutes a single class. Each glomerulus is innervated by one PN, establishing an olfactory map like the one present in vertebrates but with 1∶1 connectivity [22]–[24].

Despite the role of the larval olfactory system in patterning the adult olfactory circuit [18] and a growing number of studies using the larval system as a model olfactory network [25]–[27], almost nothing is known about its developmental origins, let alone the way in which pre- and postsynaptic neurons come together to form a functional olfactory network. Indeed, the only information we have at present concerns the precursors of the OSNs and PNs [1],[28].

Here we describe the development and regulated assembly of the larval olfactory circuit in *Drosophila* from its earliest beginnings in the embryo to functional maturity at hatching. We use a combination of genetic and dye injection techniques to determine the sequence of events leading to olfactory wiring. We combine genetic and laser ablation techniques to show that the development of the larval PNs, unlike their adult counterparts, depends on the ingrowing axons of embryonic OSNs. In addition we investigate the emergence of spontaneous patterns of activity in embryonic OSNs as they develop and show that this activity plays a role in restricting OSN glomerular territories. Our results reveal an unexpected degree of similarity between the development of the olfactory systems in vertebrates and the *Drosophila* larva.

## Results

### Developmental Events in OSNs and PNs Leading to the Wiring of the Larval Olfactory Circuit

**Figure 1:**
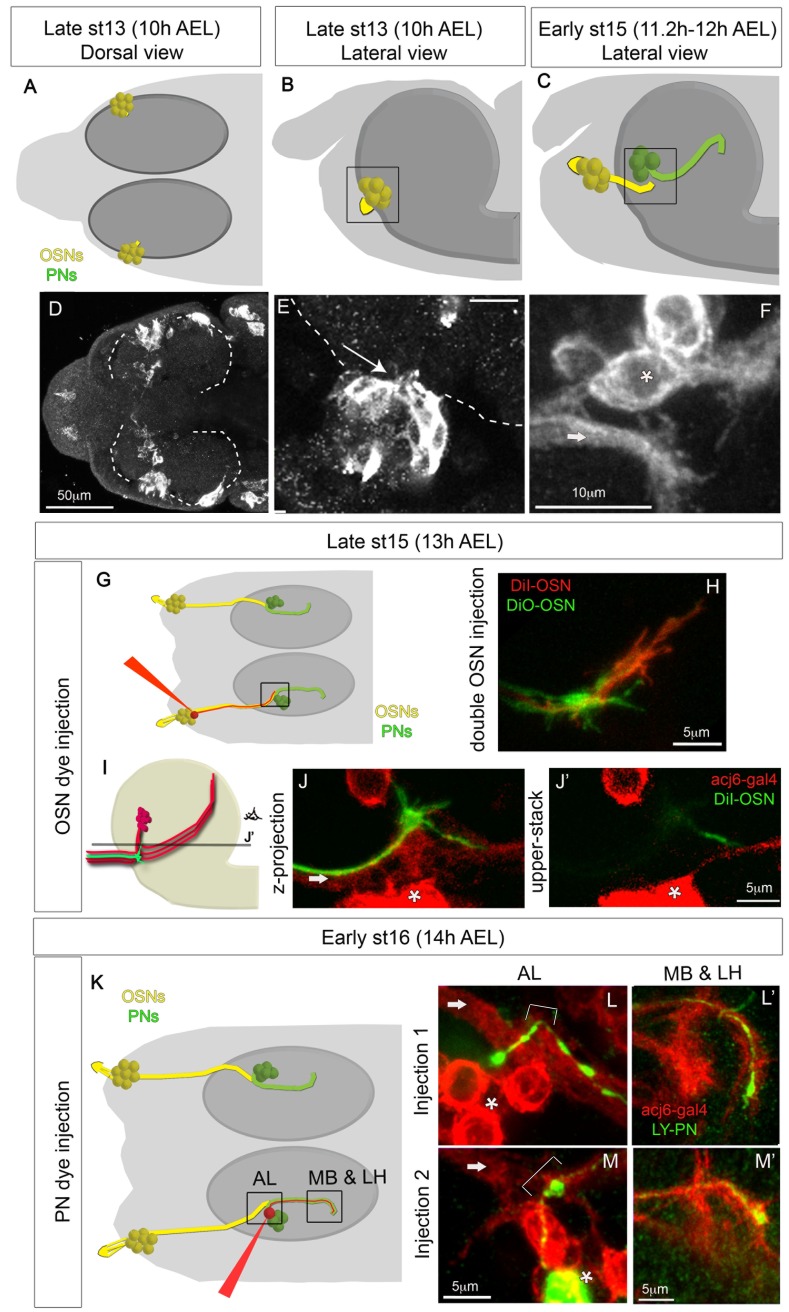
Development of OSNs and PNs between 10 h and 14 h AEL. (A–F) OSN and PN development as followed with *acj6-Gal4:UAS-CD8GFP* between 10 h and 12 h AEL, before any contact is made. In the schematics (A–C, G, and K) OSNs are yellow and PNs green. The black square in the schematics indicates the region that is being visualized in the confocal images. In the confocal stacks (E–F, J, L, and M) OSN axons are indicated with an arrow and PN cell bodies with an asterisk. (A–B and D–E) OSNs are born in direct contact with the brain (dashed line) and send short axonal projections into the brain from the very early stages (arrow in E). (F) At early stage 15 (11.2–12 h AEL) OSNs and PNs have not yet contacted each other, although they are within filopodial reach. (G–J) OSN dye injections at 13 h AEL. (G) Schematic showing the injection procedure and marking the region shown in the confocal stacks (H–J) with a black square. (H) Injection of two OSNs with different dyes showing growth cones in both OSNs. (I) Schematic showing the position of OSN axons and PNs. Both components are in red, as in the confocal pictures, showing GFP staining expressed on the acj6-Gal4 pattern. One OSN is green showing filopodia, which represents a single injected OSN as in (J and J′). OSN axons enter the brain in a ventral position respective to PN cell bodies. PN axons run ventrally towards the place of OSN terminals and then turn dorsally towards higher brain centres, making thus a U shape. Different PNs turn their axons at different dorso-ventral depths, and OSN axonal growth cones contact even the most dorsally turning PN axons. The line in (I) marked with the letter J′ represents the confocal z stack shown in (J′). (J) is a confocal z projection of what is represented in (I), while (J′) is a single z stack at the position indicated in (I). From here onwards letters with apostrophe (‘) indicate images taken from the same animal. (K–M) PN dye injections at 14 h AEL. (K) Schematic showing the injection procedure and marking the regions (AL, and MB&LH) shown in the confocal stacks (L–M). (L–M) In each example a single PN was injected (green), and all OSNs and PNs are labelled in red with *acj6-Gal4;UAS-CD8GFP*. The AL and the MB and LH regions are shown for each injected PN. At 14 h AEL PNs have already extended axons towards higher brain centres, but they have not yet sprouted dendrites at the AL. The axons in the MB and LH are still immature with growth cones. Arrows indicate OSN axons, and brackets indicate the region where the AL is forming and therefore the region where PNs will sprout dendrites.

**Figure 2:**
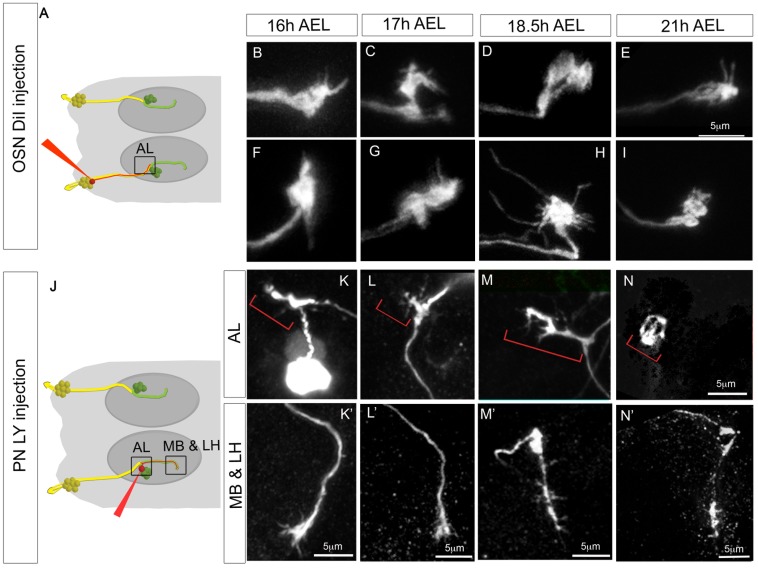
Development of OSNs and PNs between 16 h and 21 h AEL. (A–I) Development of OSNs between 16 h and 21 h AEL as revealed by dye injection. (A) Schematic of the injection procedure; as in Figure 1, the square indicates the area shown in the confocal images. Columns show injections at different developmental times. The two rows (B–E) and (F–I) each represent a different example of OSN injection. All figures are at the same scale. Terminals at 21 h AEL (E and I) appear more compact than at earlier stages. Morphologies found at 18.5 h AEL (two examples in D and H) are especially variable, with some showing long filopodia (H) or broad regions of occupancy (D). (J–N′) Development of PNs between 16 h and 21 h AEL as revealed by dye injection. (J) as (A). (K–N′) The two rows show the AL and the MB and LH regions for a representative PN injection at each developmental stage. Red brackets indicate the PN dendrites.

To study the development of the olfactory system, we used a combination of genetic labelling with the Gal4/UAS system [29] and single cell dye injection. We visualized OSNs and PNs at early stages, before any contact is made, by using *acj6-Gal4*, which is expressed in all embryonic OSNs [30] and PNs (personal observation). We find that OSNs are born in contact with the brain (Figure 1A–E), and from stage 13 (about 10 h AEL) onwards they extend short axonal projections into the brain (Figure 1E). The OSN axons elongate during development as the process of head involution [31] displaces them away from their initial lateral-ventral position towards their final more dorsal and anterior position. Within the brain, OSN axons grow a short distance until they reach the location of the future AL (Figure 1A–F). The PNs extend axons towards higher brain centres before they sprout their dendrites (Figure 1C, 1F, and 1K–M). OSN axon terminals first make contact with the most proximally located region of the PN axonal bundle at late stage 15 (about 12 h AEL) (Figure 1F and 1J). To get a detailed insight into this process, we performed single cell dye injections of OSNs and PNs at seven different developmental stages (13 h, 14 h, 15 h, 16 h, 17 h, 18.5 h, and 21 h AEL). In total we injected 73 OSNs (on average 10.42 for each stage, and a minimum of six) and 107 PNs (on average 17.83 for each stage, and a minimum of seven). At 12–13 h AEL, when OSN axon terminals contact the dendrite-less axons of PNs for the first time, all OSN terminals have prominent growth cones (Figure 1H and 1J, *n* = 11), which in 50% of the filled OSNs are still present at 14 h AEL (*n* = 10), but not at 15 h AEL or later (compare Figure 1H–J to Figure 2B–I). Injections of pairs of OSNs with two different dyes at 13 h and 14 h AEL, when filopodia are still present, reveals that even at these early stages OSN terminals occupy distinct territories, which may represent their final positions within the larval AL (Figure 1H). At 14 h AEL none of the PNs injected had sprouted dendrites yet (*n* = 14, Figure 1L–L′ and 1M–M′, and Figure S1). At 15 h AEL only 43% of the PNs injected had a dendrite (*n* = 21), while by 16 h AEL all PNs injected showed dendritic growth (*n* = 18, Figure 2K). Thus, unlike the *Drosophila* adult, in the embryo, PNs only extend dendrites after they have been extensively contacted by axonal terminations of OSNs.

We now followed the further maturation of the OSN terminals and PN dendrites and axons until hatching at 21 h AEL. OSN terminals are very variable during embryogenesis (Figure 2B–G), but by 18.5 h AEL the terminals begin to mature, they are more condensed, although they still have filopodia and overshoot their glomerular territories (Figure 2D and 2H). By hatching, terminals are less variable and more compact (Figure 2E and 2I). PN dendrites with short filopodia grow and branch in restricted glomerular-sized territories from the very beginning rather than growing in an exploratory fashion followed by pruning (Figure 2K–N). The structure of PN dendrites is very variable during development and continues to be so until hatching (Figure 2K–N). This makes it difficult to identify unequivocally when PN dendrites acquire their mature structure. The output sites of PNs, at their terminals in the MB and LH, are still immature at 16 and 17 h AEL with prominent growth cones (Figure 2K′ and 2L′). It is only at 18.5 h AEL that axons of PNs start acquiring their characteristic first instar larval morphologies, without growth cones and innervating one or two glomeruli in the MB (Figure 2M′, compare to Figure 2N′). Taken together our results suggest that 18.5 h AEL is the stage at which pre- and post-synaptic components of the larval olfactory system first begin to acquire their mature morphologies.

### OSNs Are Spontaneously Active during Development

Since larval OSNs have been reported to fire action potentials spontaneously [27], we wondered when this activity starts, and whether it plays a role in the development of the olfactory network during embryogenesis. We therefore developed a technique to record extracellularly from embryonic OSN cell bodies.

**Figure 3:**
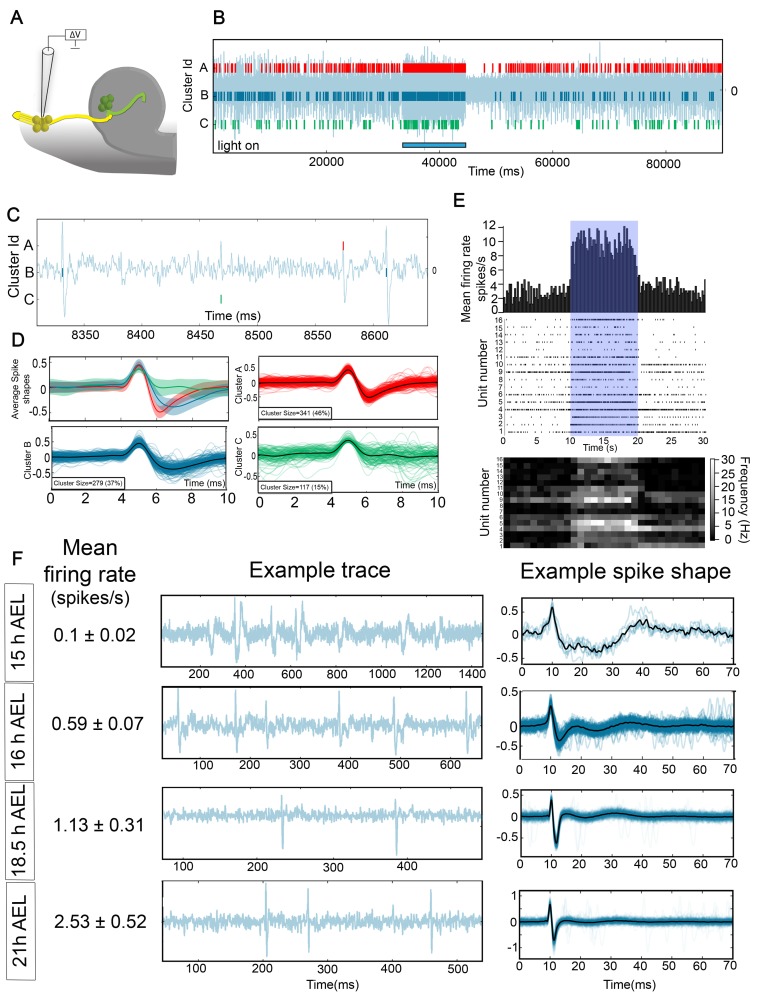
Recording from OSNs during development. (A–D) OSN recording technique. (A) Schematic showing the way in which the recordings were performed. Embryos were dissected to expose the DOG, a suction electrode was placed in contact with the DOG, and suction applied until a seal was made and action potentials were visible on the recording. (B) Example recording trace from a first instar larvae expressing ChR2 in OSNs (*Orco-Gal4;UAS-CD8GFP/UAS-ChR2*). The blue bar at the bottom indicates the period of time when the UV light was turned on to activate ChR2. In this particular recording three different units were identified using Spikepy. (C) Experimental trace from (B) showing the shape of individual spikes. (D) Clusters separated with Spikepy. Average spike shapes are plotted with their standard deviation at the top left. The other three panels are an overlay of a maximum of 250 individual spikes for each cluster, with the average spike shape plotted in black. (E) Peri-stimulus time histogram (PSTH), Raster plots, and heat maps showing the response to UV light stimulation of 16 units recorded in three different larvae in response to five different stimuli presentations in *Orco-Gal4;UAS-CD8GFP/UAS-ChR2* animals. PSTH (top panel) shows the average firing rate of the 16 units in 200 ms bins. Raster plots (middle panel) show the raw data for each of the 16 units. Heat maps (bottom panel) show for each unit the firing rate in 1 s bins. For each unit the average firing rate in the 10 s before light stimulation was significantly lower (*p*<0.001) than the average firing rate in the 10 s during the stimulation. (F) Firing rate, example trace, and example spike shape of OSN recordings at four different developmental stages.

Using this technique, we recorded simultaneously from a random sample of up to 6 of the 21 OSNs in the DOG (Figure 3A). In each recording, electrophysiological activity was allocated to individual OSNs using the newly developed spike sorting software Spikepy (http://code.google.com/p/spikepy/, Figure 3B–D, Materials and Methods). To confirm the activity we recorded was indeed originating from OSNs, we expressed the light activatable channel, Channelrhodopsin-2 (ChR-2) [32] in all OSNs (*Orco-Gal4;UAS-CD8GFP/UAS-ChR2*). We found that when OSNs were activated by exposure to 470 nm light, there was an increase in the firing rate of all units in our recordings (Figure 3B and 3E; for each individual unit the mean firing rate in the 10 s during light stimulation was significantly higher than the mean firing rate in the 10 s before light stimulation, *p*<0.001), confirming that the activity we record does indeed derive predominantly from OSNs.

We now asked when OSN activity begins by recording from the OSNs of embryos at different stages. We found that only 42% of the embryos showed OSN action potentials at 15 h AEL, even when stimulated with ChR2 (*n* = 12), while from 16 h AEL and onwards, we recorded spontaneous activity in every embryo we tested. Thus, we conclude that spontaneous spiking in OSNs begins at about 15 h AEL. Interestingly, action potentials recorded extracellularly at these early stages (15 h AEL) have a different shape, and a longer time course than the ones recorded at later stages, and the shape changes progressively over development (Figure 3F). The spontaneous firing rate is also lower at earlier stages and increases progressively as development proceeds (Figure 3F, firing rate at 15 h, 0.1±0.02 Hz, *n* = 25 units from four different embryos; 16 h, 0.59±0.07 Hz, *n* = 37 units from eight different embryos; 18.5 h, 1.13±0.31 Hz, *n* = 21 units from six different embryos; first larva: 2.53±0.52 Hz, *n* = 24 units from four different larvae).

### Developmental Changes in OSN Spontaneous Activity Patterns Depend upon Orco Function

The olfactory system of *Drosophila* larvae is thought to code for the presence of particular odours using a rate coding strategy, combined with a population code [26]. This means that in response to an odour each OSN responsive to that odour codes for its presence by changing the frequency at which it fires action potentials (rate coding), rather than by precise timing of spikes (temporal coding). Signal detection theory suggests that discriminability between the presence and absence of a stimulus (stimulus detectability) is governed by the absolute value of the difference between the mean of the spike counts with and without stimulus divided by the square root of the summed variances of these spike counts (equation 1). Thus stimulus detectability is enhanced by low variability in the spike train, because this results in low variability in spike count (low variance) over the counting window.

**Figure 4:**
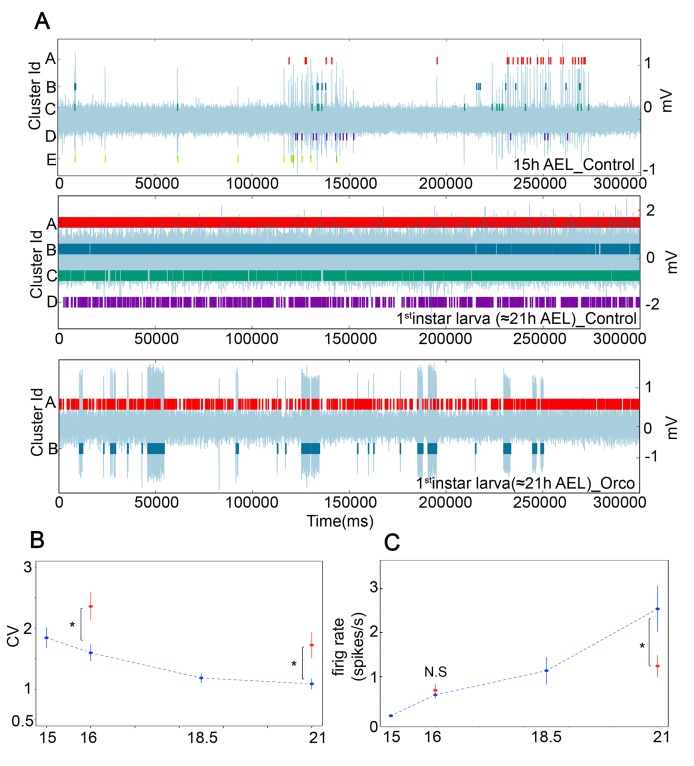
Developmental changes in OSN spontaneous activity patterns depend upon OR function. (A) Five-minute representative trace recordings overlaid with individual OSN units as identified with Spikepy. Top trace control 15 h AEL, middle trace control first instar larvae, and bottom trace Orco mutant first instar larva. OSNs at early stages (15 h and 16 h AEL) often fire in bursts, unlike OSNs at later stages (18.5 h and first instar larvae). OSNs in Orco mutants show throughout development and even in first instar larva stages bursty activity patterns. (B) Coefficient of variation of the interspike intervals (CV) at different developmental stages in controls (blue) and *Orco* mutants (red). CV is significantly higher in 16 h AEL embryos and first instar *Orco* mutants than in controls, which demonstrates that at least part of the reduction in spike train variability as development proceeds is due to OR expression. (C) Firing rate at different developmental stages in controls (blue) and *Orco* mutants (red). The firing rate at 16 h AEL is similar in *Orco* mutants and controls, but there is a significant difference between controls and *Orco* mutants in first instar larvae. (B–C) Error bars represent SEM. A single asterisk (*) indicates *p*≤0.05. *N.S.* indicates *p*>0.05.

In our data we observe that spontaneous firing patterns at early stages (15 h and 16 h AEL) are more bursty than at later stages (18.5 h AEL and L1) (Figure 4A), with inter-burst intervals (IBIs) varying between approximately 0.5 and 2 min, similar to the IBIs found in other developing systems [33]. We wondered whether variability in the spike train (and therefore discriminability) would change during the course of development. As a commonly used and straightforward measure of spike train variability, we use the coefficient of variation of interspike intervals (CV), which is defined as the ratio of the standard deviation to the mean of the interspike intervals (ISIs); thus, the lower the CV, the lower the variability in the spike train [34],[35]. We find that as development proceeds, the CV decreases progressively, with a statistically significant step between 16 h and 18.5 h AEL (CV, for 16 h = 1.59±0.13, for 18.5 h = 1.18±0.079, *p* = 0.03; Figure 4B). Thus, the pattern of OSN activity changes over development, reducing spike train variability, which in turn might serve to increase odour detectability.

Because olfactory receptors are responsible for the high spontaneous firing rate of larval OSNs [27], we reasoned that the developmental change in the pattern of spontaneous activity in OSNs might be due to the onset of OR functioning. To test this, we recorded OSN activity from *Orco* mutants at 16 h AEL (first time when all embryos showed spontaneous activity) and first instar larvae. *Orco* mutants do not traffic the specific ORs to the membrane and are therefore anosmic [36], and their spontaneous firing rate in third instar larvae and adults is diminished but not abolished [27]. As expected, the spontaneous firing rate of *Orco* mutant first instar larvae, but not 16 h AEL embryos, is reduced by half when compared to controls (firing rate, for 16h_control = 0.58±0.07 Hz, *n* = 37 units from eight different embryos; 16h_Orco_mut = 0.68±0.13 Hz, *n* = 28 units from four different embryos; *p* = 0.55, for L1_control = 2.53±0.52 Hz, *n* = 24 units from four different larvae; L1_Orco_mut = 1.23±0.25 Hz, *n* = 24 units from four different larvae; *p* = 0.03, Figure 4C). We then analyzed the CV of the ISIs and found that indeed spike train variability is significantly increased in *Orco* mutant first instar larvae, and 16 h AEL embryos, when compared with controls (16h_control = 1.59±0.13, 16h_Orco_mut = 2.35±0.22, *p* = 0.01; L1_control = 1.08±0.08, L1_Orco_mut = 1.72±0.21, *p* = 0.01; Figure 4B), and accordingly its activity patterns are more bursty (Figure 4A). We conclude that the reduction in the variability of the spike train during development requires, at least in part, Orco expression and is therefore likely to be attributable to the onset of OR function (see Discussion).

### PNs Require Presynaptic Innervation during Development for Their Survival

**Figure 5:**
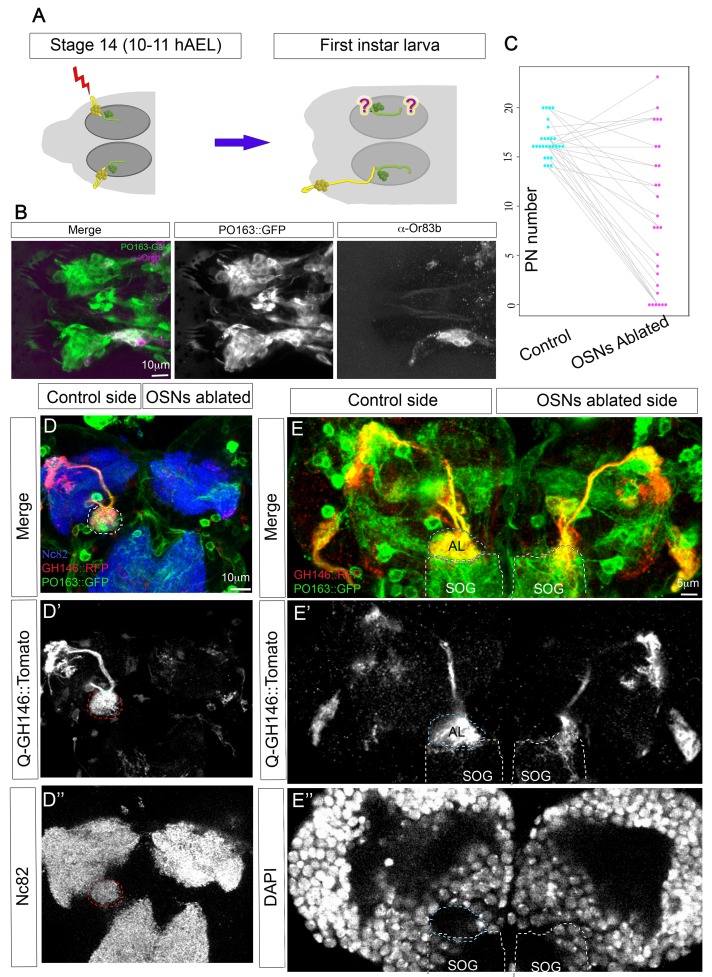
PNs require presynaptic innervation during development for their survival. (A) Schematic of the experimental approach. OSNs on one side were laser ablated at stage 14, before OSN axons contact PNs. Animals were left to develop and hatch and PN morphology was examined in mid-first instar larvae. (B) Anterior part of a first instar larva in which OSNs have been unilaterally ablated. All sensory neurons are labelled using *PO163-Gal4;UAS-CD8GFP*, including taste neurons that appear as the most anteriorly located cluster of PO163 positive cells on both sides. OSNs labelled both with PO163 in green and with α-Orco antibody (magenta) are present on the control side (bottom) but not in the ablated side (top). (C) Quantification of the OSN ablation experiments. Number of PNs in the control (blue dots) and OSN ablated side (pink dots) for each animal are linked by a grey line. (D–D″) Z projection of a brain in which OSNs were unilaterally ablated (right side). PNs visible with *GH146-QF;QUAS-mTomato* on the control side are missing on the ablated side. The AL, visualized with Nc82 antibody staining in the control side (dashed line), is absent on the ablated side. (E–E″) Z projection of a brain in which OSNs were unilaterally ablated (right side). PNs (*GH146-QF;QUAS-mTomato*) can be seen innervating the AL in the control side. Some PN of the ablated side are missing, while surviving ones are found innervating the subesophageal ganglion (SOG). The AL, visualized in the control side as a gap with DAPI staining, is missing on the ablated side (see Figure S2).

Having identified the principal steps in the morphological and physiological development of the olfactory circuit, we decided to look for regulatory mechanisms operating to ensure integrated assembly of pre- and postsynaptic elements in the antennal lobe. Since PNs only extend dendrites several hours after they have been extensively contacted by OSNs, it seemed possible that the growth of PN dendrites might be regulated by the presence of OSN terminals. To address this question, we laser-ablated all the OSNs on one side in intact embryos before the stage at which OSNs and PNs make contact (Figure 5A). We then allowed the embryos to develop until hatching and dissected them as first instar larvae. We targeted the ablations by expressing GFP in all sensory neurons with the Gal4 line PO163 [37]. The presence of the label allowed the success of the ablation to be assessed immediately after the operation and later in the first instar dissected animals. The ablations were very specific and only OSNs were ablated, while other closely positioned sensory neurons, such as taste neurons, remained intact (Figure 5B). We visualized PNs in these animals using Q-system, a Gal4-independent expression system; specifically, we used GH146-QF, which is expressed exclusively on PNs [38]. Interestingly, our experiments show that PNs require presynaptic innervation during development to survive. In 22% of cases (*n* = 27), there were no PNs on the ablated side, as compared with complete survival of all PNs on the control side, visualized with both *GH146-QF* and *PO163-Gal4*, which is fortuitously also expressed in PNs (Figure 5C and 5D–D″). In those cases where some PNs survived on the ablated side, they were found contacting other brain regions, most commonly the subesophageal ganglion (SOG), presumably due to its proximity to the AL (Figure 5E), but occasionally PNs attracted innervation from other neighbouring axon bundles (Figure S2). In every case the AL structure was completely lost from the ablated side, whether it was visualized with Nc82 antibody staining or as a conspicuous gap in DAPI labelling (Figure 5D and 5E). Thus the survival of PNs appears to require presynaptic innervation during development, but this innervation does not need to be specifically from OSNs, and other axonal terminals can also support PN survival. Interestingly, when PNs do survive, their dendrites are normally longer than controls (Figure 5E), suggesting they elongate until they find presynaptic partners, with the implication that OSNs would normally give PN dendrites a stop growth signal.

### OSN Activity Is Not Required for PN Survival, But It Influences Dendritic Growth

**Figure 6:**
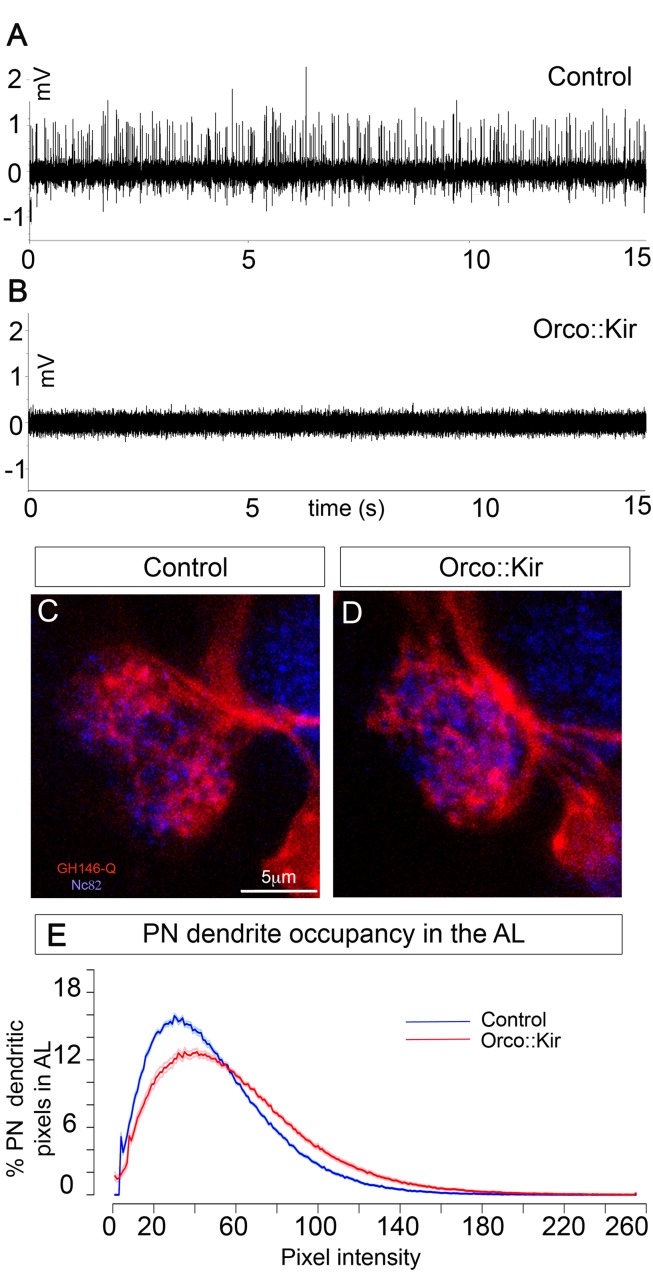
OSN activity is not required for PN survival, but it influences dendritic growth. (A, B) Representative recordings of control embryos (A) and Orco-Gal4:UAS-Kir2.1 (B). (C, D) PN dendrites (*GH146-QF;QUAS-mTomato*, red) innervating the AL (Nc82 antibody, blue) in a control (A) and where OSN spontaneous activity has been blocked by expressing Kir2.1 in all OSNs using *Orco-Gal4* (B). (E) PN dendrite occupancy in the AL as quantified by the pixel intensity distributions of PN dendrites within the AL volume in controls (blue) and Orco::Kir (red). In Orco::Kir embryos PN dendrites occupy a slightly but significantly larger area in the AL with respect to controls, as visualized by the shift in the pixel intensity distribution towards higher intensities in these embryos. The solid dark line represents the mean, and the light surrounding lines represent ± s.e.m. (*p*<0.0001, Kolmogorov-Smirnov test).

Our recordings from developing OSNs show that spontaneous activity starts at the time of PN dendrite extension (15 h AEL). This together with the finding that PNs require presynaptic innervation during development for survival suggested that PN dendrite development or survival might depend on the spontaneous firing of OSNs. We therefore silenced OSNs by expressing *UAS-Kir2.1* using the *Orco-Gal4* line, which is expressed in all OSNs before the onset of spontaneous activity (Figure S3). Expression of Kir2.1 has been shown to silence several types of *Drosophila* neurons [39],[40], and when expressed in adult OSNs, all odour responses are abolished [36], but its use in larval OSNs has not been reported. We therefore tested the effects of our manipulation by recording extracellularly as described above from control first instar larvae (*Orco-Gal4;UAS-GFP*) and larvae expressing Kir2.1 in OSNs (*Orco-Gal4;UAS-Kir2.1*). We found that in eight out of eight control larvae we could record action potentials. However, as expected, action potentials could not be recorded from any of eight Kir-expressing animals (Figure 6A and B, Materials and Methods). We then examined PNs using *GH146-QF;QUAS-mTomato*, in first instar larvae in which OSNs had been silenced by Kir expression. We found that the gross morphology of PNs in these larvae appeared normal when compared to controls (Figure 6C and D). However, quantification of PN dendritic occupancy within the AL as quantified by pixel intensity plots (see Materials and Methods) revealed a small but significant (Figure 6E, *p*<0.0001, *n* = 12) increase in PN dendrite occupancy within the AL when all OSNs had been silenced, as compared to controls. Thus, we conclude that OSN activity is dispensable for PN survival, dendrite extension, and maintenance and that the requirement for innervation that we have demonstrated is therefore likely to be contact mediated and activity independent. However, we demonstrate that in the absence of OSN activity, PN dendrites overgrow, which supports our previous observation that OSNs provide a stop growth signal to PN dendrites, probably through both contact and activity-dependent interactions.

### OSN Activity Is Essential for the Appropriate Wiring of the Olfactory Circuit

**Figure 7:**
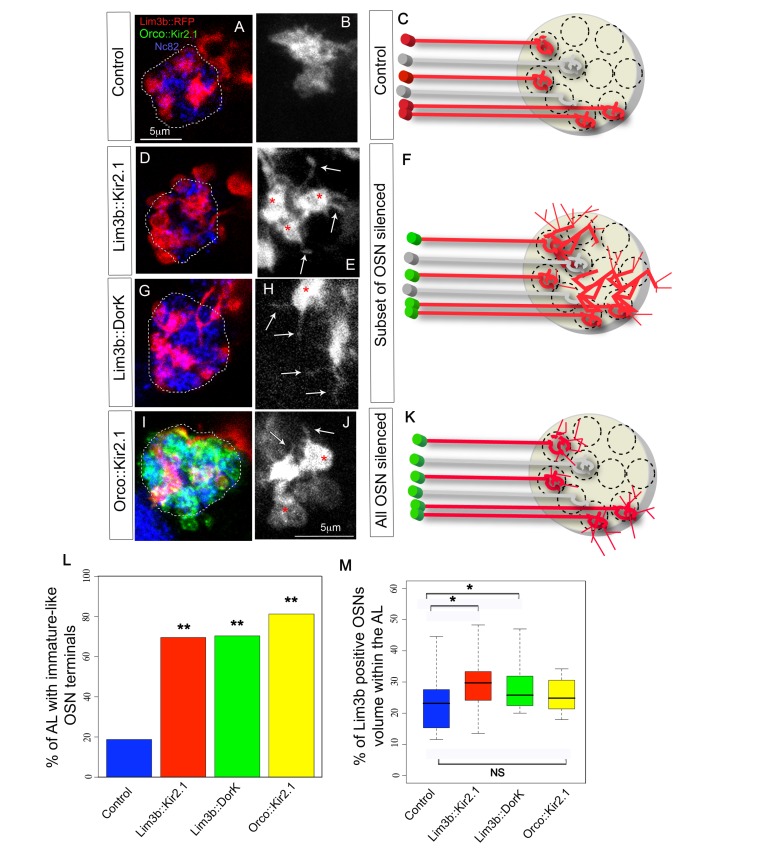
Effects of silencing a subset or all OSNs on OSN terminal arborizations. (A–K) Confocal z projections and schematics of four OSN terminals (*Lim3b-Gal4;UAS-mRFP*, red in all except B, E, H, and J where it is white) in the AL (Nc82 staining, dashed line in A, D, G, and I, and yellow circle in C, F, and K) of 21 h AEL animals, in control (A–C), Lim3b::Kir2.1 (D–F), Lim3b::DorK (G–H), and Orco::Kir2.1 (I–K). (B, E, H, and J) show some of these four OSN terminals at a higher magnification and without Nc82 counter-staining to facilitate the visualization of OSN terminal morphologies under each of the experimental conditions. In (I) green staining is *Orco-LexAOp;LexA-Kir2.1EGFP*. In (C, F, and K) red OSNs represent the four Lim3b-Gal4 positive OSNs, and the grey OSNs represent the rest of the OSNs that we are not visualizing (only two out of the 17 non-visualized OSNs are represented in the schematic). The green cell bodies mean that Kir2.1 (or DorK) is being expressed in those cells. In control animals each OSN terminal is restricted to a single glomerulus and no filopodia are observed (A–C; in A the four Lim3b positive terminals are shown, and in B only two OSN terminals are shown). When a subset of four OSNs are hyperpolarized by expressing either Kir2.1 (D–F) or DorK (G–F), these OSNs expand and occupy neighbouring glomeruli (D and G) and have immature-like morphologies (red asterisks) with filopodial projections (white arrows). When all OSNs are hyperpolarized (I–K), OSNs remain more restricted to glomerular-sized territories (I), although they also have immature-like morphologies with filopodia (J). (L) Quantification of immature-like morphologies at 21 h AEL in control (blue), Lim3b::Kir2.1 (red), Lim3b::DorK (green), and Orco::Kir2.1 (yellow). Bar plot indicates the percentage of ALs with axons with immature-like morphologies. Two asterisks (**) indicate *p*≤0.01 when compared to controls. (M) Quantification of occupancy of the four *Lim3b-Gal4* terminals within the AL volume (defined by the Nc82 antibody staining contour) at 21 h AEL in control (blue), Lim3b::Kir2.1 (red), Lim3b::DorK (green), and Orco::Kir2.1 (yellow). Box plots show the median of the distribution (middle line), the 75th percentile (upper limit of box), and 25th percentile (lower limit of box). Whiskers indicate the highest and lowest value of each experimental group. A single asterisk (*) indicates *p*≤0.05. *N.S* indicates *p*>0.05 (see Figure S3).

We next investigated whether spontaneous activity in embryonic OSNs might have a more subtle role in the wiring of the olfactory circuit. We sought to answer this question by genetically silencing and visualizing a subset of OSNs. Because we found published OSN lines that are specific for a subset of OSNs, such as OR specific lines, only begin to be expressed around 18.5 h AEL (Figure S3), we screened for new Gal4 lines that would be expressed in subsets of OSNs from earlier stages. We find that *Lim3b-Gal4* is expressed reliably in four of the 21 larval OSNs from 17 h to 21 h AEL (Figure S4), with an onset of expression in two of these OSNs at 16 h AEL. We used this line to drive expression of a new *UAS-myr-mRFP* insertion line [41], which is brighter than previously available lines, and thus allows us to visualize fine neuronal processes. We crossed this *Lim3b-Gal4;UAS-mRFP* line with *UAS-Kir2.1* or *UAS-DorK* to silence the four OSNs. DorK (*Drosophila* open rectifier K channel) has previously been shown to silence *Drosophila* neurons in a similar way to Kir2.1 [42], and we decided to use it as an alternative and independent way to silence OSNs. We find that the silenced OSNs in first instar larvae have the immature-like morphologies with broad axonal terminals and multiple filopodia that are more characteristic of earlier developmental stages than newly hatched larvae (Figure 7E and 7H). Quantification of ALs with this phenotype in control and experimental animals showed that the difference is significant (percentage of AL with immature OSN terminals: control = 19%, *n* = 16; Lim3b::Kir2.1 = 69%, *n* = 23; Lim3b::DorK = 70%, *n* = 27; *p*(control, Lim3b::Kir2.1) = 0.003; *p*(control, Lim3b::DorK) = 0.001, Figure 7A–K and 7L). Silenced terminals also appeared to be expanded within the AL when compared to controls, occasionally even extending beyond the synaptic region of the AL (Figure 7D and 7F). We reconstructed the volumes of the AL and OSN terminals (see Materials and Methods) and found that silenced OSNs indeed occupied a larger percentage of the AL synaptic volume than controls (*p*(control-*kir*) = 0.02; *p*(control-*DorK*) = 0.04; Figure 7M). These data suggest that spontaneous activity of OSNs is essential for them to develop normal axon terminals and that its absence either triggers or fails to suppress an exploratory growth programme.

Our analysis to this point has been restricted to the role of OSN spontaneous activity under competitive conditions, in which only four of the 21 OSNs are silenced while the rest have normal levels of activity. To test for possible activity-dependent interactions among neighbouring OSN terminals, we documented the morphological and volumetric characteristics of the same four Lim3b positive terminals when all OSNs had been silenced. To achieve this, we generated a *LexA-Kir2.1* line that when combined with *Orco-LexAOp* [43] silences all OSNs, leaving the Gal4/UAS system available for visualization of the same OSNs that we analyzed before (Figure 7I). Under these conditions, when activity is blocked in all OSNs, the terminals of the four Lim3b positive OSNs remain immature in the first instar larva (*p*(control_*Orco::Kir*) = 0.001; *n*(*Orco::Kir*) = 16; Figure 7I–J and 7L), comparable to the condition where all other OSNs are unaffected. We therefore conclude that this immature phenotype is cell-autonomous and independent of interactions between neighbouring terminals. However, OSNs that develop in an AL in which all OSN activity has been blocked occupy a volume within the AL that is not significantly different from that of controls (*p*(control_*Orco:: Kir*) = 0.25, Figure 7M). Thus acquisition of a mature morphology by OSN terminals requires spontaneous activity in the cells concerned, regardless of whether neighbouring cells are active or not. However, the restriction of terminal volumes within the antennal lobe is a competitive process in which silent endings appear to have a growth advantage over neighbouring active terminals.

## Discussion

A striking feature of olfactory system organization is the conserved arrangement of OSN terminals and uniglomerular PNs into an odotopic glomerular map. Previous studies lead to the conclusion that the sequence of events and developmental mechanisms patterning connectivity among OSNs and PNs in vertebrates and in insects are radically different. However, most studies of the development of the olfactory network in insects have focused on adult development. Here we uncover developmental events and mechanisms leading to the embryonic assembly of the *Drosophila* olfactory network from the beginning, before contacts are made, until functional maturity at hatching. We find that afferent ingrowth pioneers AL development and that contact and activity-dependent interactions among the components of the circuit are essential for appropriate patterning of connectivity in the larval AL. Our study provides insights into axon-to-dendrite and axon-to-axon interactions in neural circuit assembly and reveals an unexpected degree of similarity with other embryonically developing vertebrate olfactory systems. Furthermore, we provide the first systematic study of the onset and developmental maturation of normal patterns of spontaneous activity in OSNs. Below we discuss the implications of our findings in the context of general principles of neural network development and more specifically with a focus on the development of connectivity in olfactory circuits.

### PNs Require OSNs for the Adequate Development of the AL

A key finding in this study is the interdependence of OSNs and PNs for the proper development of the larval AL. Although at early stages of embryogenesis OSN and PN axons approach the site of the future AL independently of each other, once PN dendrites penetrate the emerging AL, interactions with OSN regulate the patterning of connectivity.

Embryonic development of the *Drosophila* AL begins with OSN terminals targeting distinct territories that probably represent the origins of AL glomeruli. At this stage PN axons turn away from this site and continue growing towards higher brain centres. By the time that growth cones of OSN axons contact the proximal region of PNs axons, the PNs have not yet extended any dendrites. Hours later, PN extend dendrites directed towards particular territories within the emerging AL, possibly guided by the same cues that direct OSN terminal targeting. The early arrival of OSNs in the future region of the AL before PN dendrite extension suggested a possible role for OSNs in the development of the AL. Indeed, we found that PNs require presynaptic innervation for their survival, although innervation does not necessarily have to come from OSNs. Additionally, there is no specific requirement for OSN terminals in promoting sprouting of PN dendrites since in the absence of OSNs, surviving PNs have dendrites. These dendrites are normally longer than controls, suggesting they elongate until they find presynaptic partners, with the implication that OSNs normally give PN dendrites a stop growth signal. We show that this effect is both contact and activity dependent, because PNs in animals where all OSNs had been silenced have overgrown dendrites that do not extend beyond the AL. A similar effect has been found in the dendrites of motorneurons in *Drosophila* embryos, where the removal of presynaptic terminals induces an overgrowth of postsynaptic motorneuron dendrites that anticipates the dendritic overgrowth induced by the lack of pre-synaptic activity at later developmental stages [44].

Independently of whether PNs survive or not, in all cases the AL is lost when OSNs are ablated. Loss of the AL has also occurred on an evolutionary scale in terrestrial isopods, which in the process of colonising the land have secondarily lost their olfactory sensilla in the main olfactory appendage, together with the corresponding olfactory deutocerebral structures (second neuromere of the supraesophageal ganglion where the olfactory lobe is located). Furthermore, in some species the tritocerebrum (posteriorly adjacent neuromere to the deutocerebrum) seems to have acquired additional neuropile structures [45]. Our findings show that there is an interdependence in the development of the *Drosophila* embryonic olfactory system that results in the loss of deutocerebral olfactory structures (the AL) in response to the ablation of OSNs. At the same time the finding of occasional ectopic tritocerebral and subesophageal innervation of PNs indicates a possible developmental route for the evolutionary acquisition of additional tritocerebral structures.

Our results contrast with previous studies in adult *Drosophila*, which show that PNs pioneer development of the adult AL independently from adult OSN development. Why is development of the olfactory system in *Drosophila* different during embryogenesis and metamorphosis? Interestingly, experiments in other embryonically developing olfactory systems, in both vertebrates and invertebrates, also demonstrate an essential role for OSN ingrowth in the development of their first olfactory centres. Experiments in *Xenopus* where OSNs were removed unilaterally at early embryonic stages showed that an olfactory bulb fails to develop on the ablated side, but is present on the control side [46]. Similarly, an experiment in cockroaches where most, but not all, OSNs were unilaterally removed during embryogenesis before they innervate the AL showed that the deafferented lobe was severely disrupted, its characteristic glomeruli were missing, and it was markedly reduced in volume. Furthermore, as with our findings, PNs in these partially deafferented lobes were sparsely branched and had elongated dendrites instead of their characteristic uniglomerular tufts [47]. In contrast, when OSNs were ablated early in adult development in insects (*Manduca* [48] and *Drosophila* adult [17]) an AL still formed, and PN dendrites arborized in their glomerular territories. We conclude that the differences we find in the development of the *Drosophila* larval and adult olfactory systems probably arise from fundamental differences between embryonic development and metamorphosis. In embryos (vertebrate or *Drosophila*) there is no preexisting network to guide development, whereas during metamorphosis the adult olfactory system makes use of cues derived from the larval olfactory system [18]. Thus its wiring seems to rely more on external cues and less on interactions among its network components than the wiring of the larval network.

### Spontaneous Activity Patterns during Development

Our method allows spontaneous activity to be recorded from OSNs developing in vivo in the *Drosophila* embryo. Although it has been assumed that OSNs in mice and insects may be active during development (see below) [19],[21], and there is a previous report of activity recorded from the antennal nerve of *Manduca* during adult development [49], ours is, to our knowledge, the first systematic description of the onset and developmental maturation of normal patterns of spontaneous activity in OSNs.

Our results reveal three important features about the development of activity patterns in OSNs:

As in other developing systems [50],[51], the earliest action potentials generated by OSNs are different from mature ones, with smaller amplitude and a longer duration. Such changes in spike shape seem to be a general feature of emerging activity as ionic conductances are acquired and mature.

At early stages we record intermittent bursts of activity in the OSNs. Activity patterns that consist of spontaneous bursts are common to many developing neural networks, including the auditory [52], visual [53], motor [54],[55], and olfactory systems (this study and [50] and their time course is remarkably similar across different neural systems, with inter-burst intervals varying between 0.5 and 2 mi [33] like those we report here). Such activity may be an inevitable consequence of cells acquiring mature excitable properties, but it is also possible that the generality of these activity patterns, and the diversity of mechanisms by which they are generated and terminated, is an indication of an essential and significant role in the development of neural networks [33].

As development proceeds, variability of the spike train diminishes, which is predicted according to information theory to increase signal (odour) detectability. A previous in vitro study of locust frontal ganglion neurons showed that there is a transient period during the wiring process when activity is irregular, but as the network matures, regularity increases [56]. As far as we know, ours is the first direct statistical analysis of the transition from immature to mature spike-trains in vivo and allows us to suggest that the coding capabilities of the network improve as it develops. It seems likely that a change towards patterns that would be expected to increase signal detectability, and thus network functionality, would be a general feature in neural networks as they mature.

The mechanisms by which this immature activity is generated, shaped, and terminated vary from system to system [33]. In the embryonic OSNs, the transition from irregular spike-trains to continuous discharge may require the expression of olfactory receptors (OR), because in larvae mutant for the co-receptor Orco, necessary for OR function, this transition does not occur normally. Since Orco is expressed before the onset of spontaneous activity (Figure S3), we suggest that the change in the pattern of OSN spontaneous activity is likely to be driven, at least in part, by the onset and level of expression of specific ORs. However, this might not be the only factor shaping spontaneous activity patterns over development, and other factors such as expression of other ion channels may also play a role. This might explain why 16 h AEL Orco mutants have indistinguishable levels of activity when compared with controls, yet the variability in their spike train is significantly increased.

### Role of Spontaneous Activity in the Wiring of Olfactory Circuits

Previous studies have suggested that spontaneous activity is essential for the normal development of vertebrate OSNs, but that there is no such requirement in insects [19],[21],[49],[57].

However, we find that there is a role for OSN activity in the development of the larval olfactory network. OSN activity regulates the morphology of OSN terminals independently of activity in neighbouring axons, and without activity terminals appear immature and occupy larger territories. This is similar to what has been described in zebrafish and mouse OSN terminals devoid of activity [19],[57]. There is also a report of a similar phenotype found in the AL of third instar *Drosophila* larvae after synaptic release was blocked in a large subset of OSNs [58]. Our results show that while immature terminal morphology is a cell autonomous phenotype that is independent of activity levels in neighbouring OSN axons, the expansion of OSN terminals is limited by interactions among the OSN terminals. Interestingly a similar process has been found to regulate the morphology and terminal expansion of retinotectal axons [59]. Thus the control of axonal terminal extension via activity-dependent interactions may be a general process in the wiring of nervous systems. The nature of inter-axonal interactions that limit terminal growth remains unknown and is one example of how future work using amenable experimental systems such as the one provided by the larval olfactory network in *Drosophila* larvae may reveal general mechanisms operating during the assembly of neural circuitry.

## Materials and Methods

### Embryo Collection and Fly Stocks

Eggs were collected from flies kept on apple juice agar supplemented with yeast paste and maintained at 25°C. For ChR2 expressing flies, we supplemented the yeast paste with all-trans retinal (Sigma-Aldrich) to a final concentration of 1 mM. Fly stocks and recombinant chromosomes were generated using standard procedures.

### Whole Mount Staining

For embryonic stages before 13 h AEL, flies were left to lay eggs on agar plates for 14 h at 25°C and whole mount stainings were performed. Whole mount immunostainings followed standard protocols. Primary antibodies used were: goat anti-GFP 1∶500 Abcam and mouse 22c10 1∶20 DSHB (Developmental Studies Hybridoma Bank, USA) secondary antibodies were Alexa 488 and Cy3 conjugated (1∶800, Invitrogen and Jackson Lab, respectively). Specimens were cleared and mounted in Vectashield (Vector Laboratories) under n°1 coverglasses, and for the different orientations they were rotated under the coverglass before the confocal scans.

### Staging, Staining, and Dye Injection

Embryos were dechorionated with bleach for 5 min and selected during two short windows, either at the three-part gut stage (13 h AEL) or when their main dorsal tracheae begin to fill with air (18.5 h AEL). These embryos were placed on a 25°C agar plate in an incubator for the number of hours required for each time point. PNs were filled with Lucifer Yellow as described in [44]. Primary antibodies used were: rabbit anti-Lucifer Yellow 1∶1,000, Invitrogen; goat anti-GFP 1∶1600, and AbCam; mouse Nc82 [60], 1∶50, DSHB. Secondary antibodies were Alexa 488, Cy3, and Cy5 conjugated (1∶800, Invitrogen and Jackson Lab, respectively). OSNs were filled with the lipophilic tracer dyes DiI and DiO, because the limited diffusion of LY did not allow visualization of the terminal axons of OSN in the AL. Embryos of the appropriate stage were dissected on poly-Lysine-coated coverslips up to 15 h AEL and on Sylgard-coated coverslips for embryos older than 15 h AEL as in [41], but opening the front of the animal to get access to the OSNs. Injections proceeded as described for Lucifer yellow injections, but OSNs were filled intracellulary with DiI or DiO dissolved in dry EtOH (2 mg/ml). The dye was allowed to diffuse for 2–4 h at 4°C, and specimens were mounted as described for PN preparations.

### OSN Ablation

For ablation experiments, embryos were dechoronionated with bleach for 5 min and selected at stage 14 based on features as described in [31] and OSNs were visualized under a 60× dipping lens with a LSR ultraview scan head mounted on a Leica DM6000B spinning disk microscope. OSNs were ablated with a Micropoint laser ablation system from Spectra-Physics (USA) mounted on this microscope and using a computer interface from Metamorph software (molecular devices). Once OSNs were ablated, embryos were placed in individual apple juice agar plates and left to develop for 24 h. Brain dissections and antibody stainings were as for the Lucifer Yellow injections. No antibody was used to visualize RFP fluorescence, and the images show native RFP fluorescence after fixation and antibody staining in the other two channels.

### Confocal Imaging, Data Acquisition, and Analysis

Images were collected using a Leica SP5 confocal laser scanning microscope. Image z-stacks were processed using ImageJ 1.39 s software (U.S National Institute of Health, Bethesda, Maryland, USA, http://rsbweb.nih.gov/ij/), and figures were generated using Photoshop CS2 (Adobe Systems, San Jose, CA). For calculating AL and OSN volumes in Figure 7, the “Segmentation editor” plugin of ImageJ was used. To calculate the data in Figure 6E, images were imported in Amira (http://www.amira.com/), and AL volumes were marked using the segmentation editor, and used as a mask in the PN dendrite channel, where intensity histograms using a bin number of 256 were calculated. These data were exported to Excel where intensity histograms were normalized for total pixel number for every stack and subsequently exported to “R project” (R Foundation for Statistical Computing, Vienna, Austria, 2005. http://R-project.org) for plotting. For delimiting the AL volume the contour of the Nc82 staining of the AL was used.

### Generation of the LexA-Kir2.1 Transgene

The pLOT-EGFP-Kir construct was created by amplifying the EGFP-Kir cDNA from pUAST-EGFP-Kir vector [39] by Polymerase Chain Reaction (PCR) using the following Gateway primers: attB1-ATG GTG AGC AAG GGC GAG GAG CTG T and attB2-TCA TAT CTC CGA TTC TCG CCG TAA G. The PCR product was introduced into pDONRTM221 (Invitrogen) via Gateway cloning to create the pEntry-vector. In the subsequent Gateway cloning reaction, this vector was combined with the pLOT-W vector [61] to fuse the EGFP-Kir channel downstream of the LexA operon. DNA was purified using a Qiagen Midi Kit and transgenic lines were generated by BestGene Inc. (Chino Hills, CA, USA).

### In Vivo Electrophysiology: Extracellular Multiunit Recordings

Embryos of the appropriate stage were dissected as for OSN dye injections in physiological saline composed of (in mM): 135 NaCl, 5 KCl, 5 CaCl_2_-2H_2_O, 4 MgCl_2_-6H_2_O, 5 TES (2-[[1,3-dihydroxy-2-(hydroxymethyl)propan-2-yl]amino]ethanesulfonic acid), 36 Sucrose, adjusted to pH 7.15 with NaOH. Borosilicate glass capillaries were pulled with a Sutter Instruments P-87 puller and fire polished to achieve a final tip of approximately 5 µm. The recording electrodes were back filled with physiological saline. The recording electrode was placed close to the DOG under an Olympus BX51WI microscope with a 60× water immersion objective using a hydraulic micromanipulator (Narishige), and suction was applied with a syringe until a seal was obtained and action potentials could be recorded. Normally between one and eight cells were sucked into the pipette. A wire in the bath acted as reference electrode. Voltage signals were amplified with a differential AC amplifier (AM-Systems, Sequim, WA). Signals were low-pass filtered at 10 KHz and digitalized at 20 KHz. Data were digitized using a Power Lab 4/30 and recorded with LabChart5 (AD instruments).

### Electrophysiology Extracellular Multiunit Analysis

Data acquired with LabChart5 were exported as MATLAB files. Recordings were divided into 5 min segments and imported into Spikepy, an open-source spike sorting software (http://code.google.com/p/spikepy/). At least one segment of 5 min was analyzed per animal. When recordings were very long, a maximum of four representative segments were analyzed. Units were separated using Spikepy with procedures that were adopted with the aim of reliably distinguishing cells rather than picking up the maximum number of cells. Briefly, the software filters the data to remove part of the baseline noise, applies an amplitude threshold for detecting the spikes, then extracts spike features for spike sorting using the full spike shape, and then clusters the spikes using the K-means method. Thus, Spikepy discriminates spikes based on shape and amplitude. It then outputs the results as graphs as shown in Figures 3 and 4, and as a MATLAB file containing information on each cluster, among other parameters. MATLAB files generated by Spikepy were opened in MATLAB, and firing rates, PSTH, and CVs were calculated.

For experiments referring to the absence of action potentials in Orco-Gal4::UAS-Kir2.1 animals, recordings were performed alternatively from control and experimental animals (*n* = 8 for each condition). Our success rate of recording action potentials in control animals was of 100%, however in the Orco::Kir2.1 animals, no action potentials could be recorded, even after repeated trials.

### Statistical Analysis

Data were analyzed and plotted using “R project” (R Foundation for Statistical Computing, Vienna, Austria, 2005; http://R-project.org). Data were analyzed statistically using the Shapiro-Wilk test to assess for normality followed by a Student's *t* test or a Wilcoxon rank-sum test as appropriate. The exceptions are the data in Figure 7L, which are categorical and therefore analyzed with Fisher's exact test, and data in Figure 6E, which were analyzed using a Kolmogorov-Smirnov test:
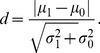
(1)


## Supporting Information

Figure S1 Related to Figure 1. At 14 h AEL PN dendrites are absent. Further examples of injections of PNs at 14 h AEL that clearly show the absence of dendrites in PNs at this stage. The arrow indicates the axons of OSNs, and the empty circle shows the region of the forming AL, where PN dendrites will sprout.
(TIF)

Figure S2 Related to Figure 5. PN dendrites attract ectopic innervation when OSNs are killed early in embryogenesis. (A–A′″) Z projection of dorsal z stacks. The AL is visible on the control side (dashed line), with PN dendrites innervating it, and as a gap in DAPI staining, however on the ablated side, due to a combination of a different mounting orientation of that brain lobe, and to the fact that PN dendrites in the ablated side have grown more ventrally to “find” presynaptic innervation, only the axons and some cell bodies of PNs are visible, but not the dendrites. (B–B′″) Z projection of ventral z stacks. PN dendrites in the OSN ablated side have attracted presynaptic innervation from some nearby cell bodies (asterisk), and an axonal fascicle labelled in the PO163 pattern with GFP that on the control side runs from the brain lobes into the SOG (green fascicle surrounded by a white circle) and that on the ablated side has been “sequestered” by PN dendrites.
(TIF)

Figure S3 Related to Figure 7. Onset of expression of receptor Gal4 lines. (A) Orco is expressed from early stages; here expression at 14 h AEL is shown with anti-Orco antibody staining. OSNs are surrounded by a white circle. (B–C) Orco Gal4 begins to be expressed at 13 h AEL (B), but GFP cannot be detected in the terminals in the AL until 14 h AEL (C). OSNs are surrounded by a white circle, and AL is enclosed in a white square. (D–F) Or94b-Gal4, a Gal4 for a specific OR, only begins to show expression in the AL at 18.5 h AEL. Half of the screened embryos did not show any expression at 18.5 h AEL (D), but the other half did show a faint expression (E). At hatching time the Gal4 line is reliably expressed in one OSN terminal (F).
(TIF)

Figure S4 Related to Figure 7. Lim3b Gal4 is expressed in four OSNs. Four cells that express Lim3b Gal4 (red) overlap with anti-Orco antibody staining (blue) that labels all OSNs. Two of the Lim3b positive OSNs have their cell bodies in a dorsal position and send their dendrites together to a common sensillum of the DO. The other two Lim3b positive OSNs are situated in a more ventral position within the DOG and send their dendrites together to another common DO sensillum. The samples are also labelled with Orco-LexA driving mCD8GFP (green), and this line is expressed in all OSNs and marks the contour of the AL, where four glomeruli are labelled by the four Lim3b positive OSNs in red. Scale bar, 5 mm.
(TIF)

## Abbreviations

AL: antennal lobe
OSN: olfactory sensory neuron
PN: projection neuron
OR: olfactory receptor
