# Supplementary figures and images for: Embryonic Origin of Olfactory Circuitry in Drosophila: Contact and Activity-Mediated Interactions Pattern Connectivity in the Antennal Lobe
Source: PLoS Biol. 2012 Oct 2;10(10):e1001400. doi: 10.1371/journal.pbio.1001400 (PMC3462790; doi:10.1371/journal.pbio.1001400)

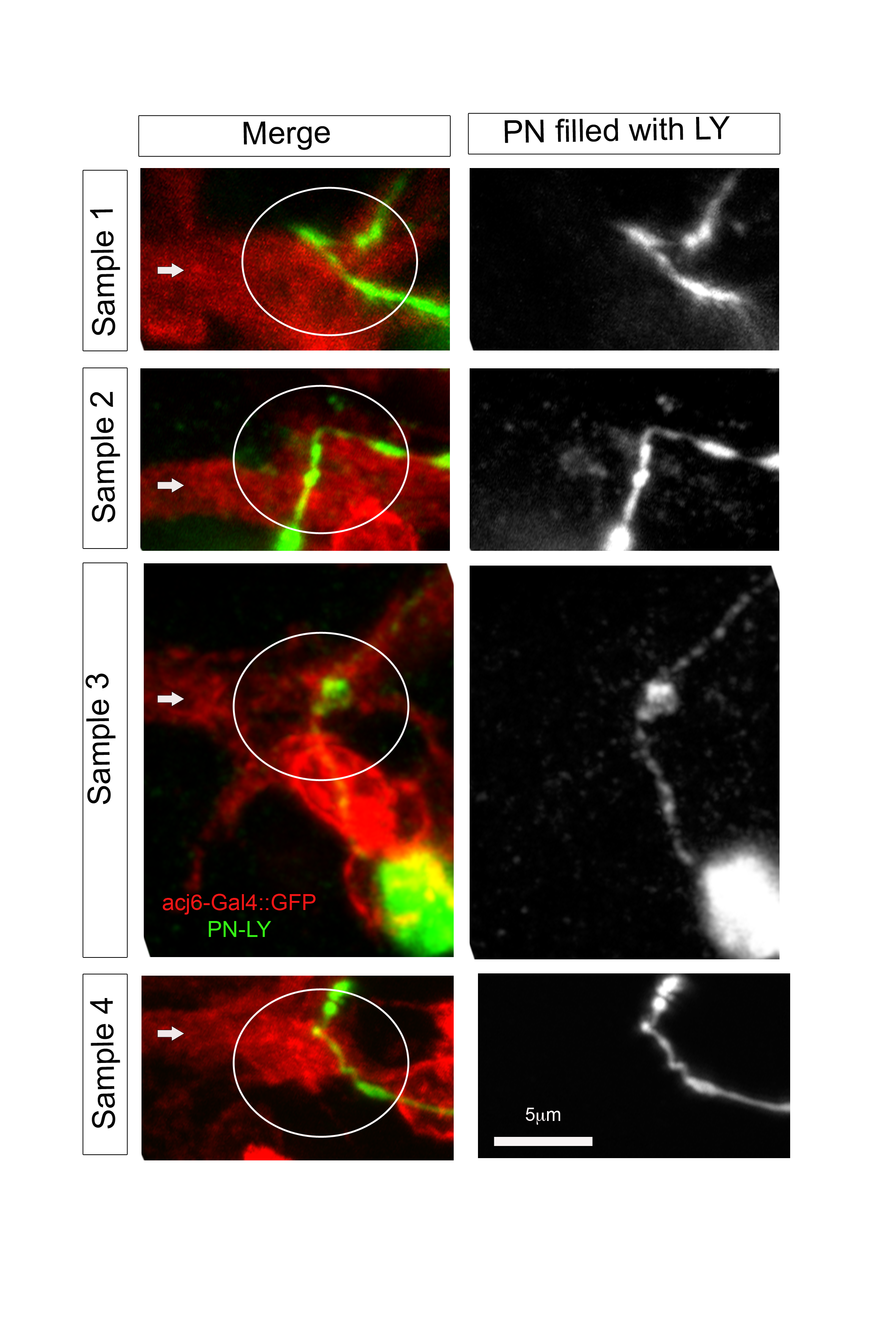

Supplement: Figure S1 — Related to Figure 1. At 14 h AEL PN dendrites are absent. Further examples of injections of PNs at 14 h AEL that clearly show the absence of dendrites in PNs at this stage. The arrow indicates the axons of OSNs, and the empty circle shows the region of the forming AL, where PN dendrites will sprout. (TIF) [file pbio.1001400.s001.tif]

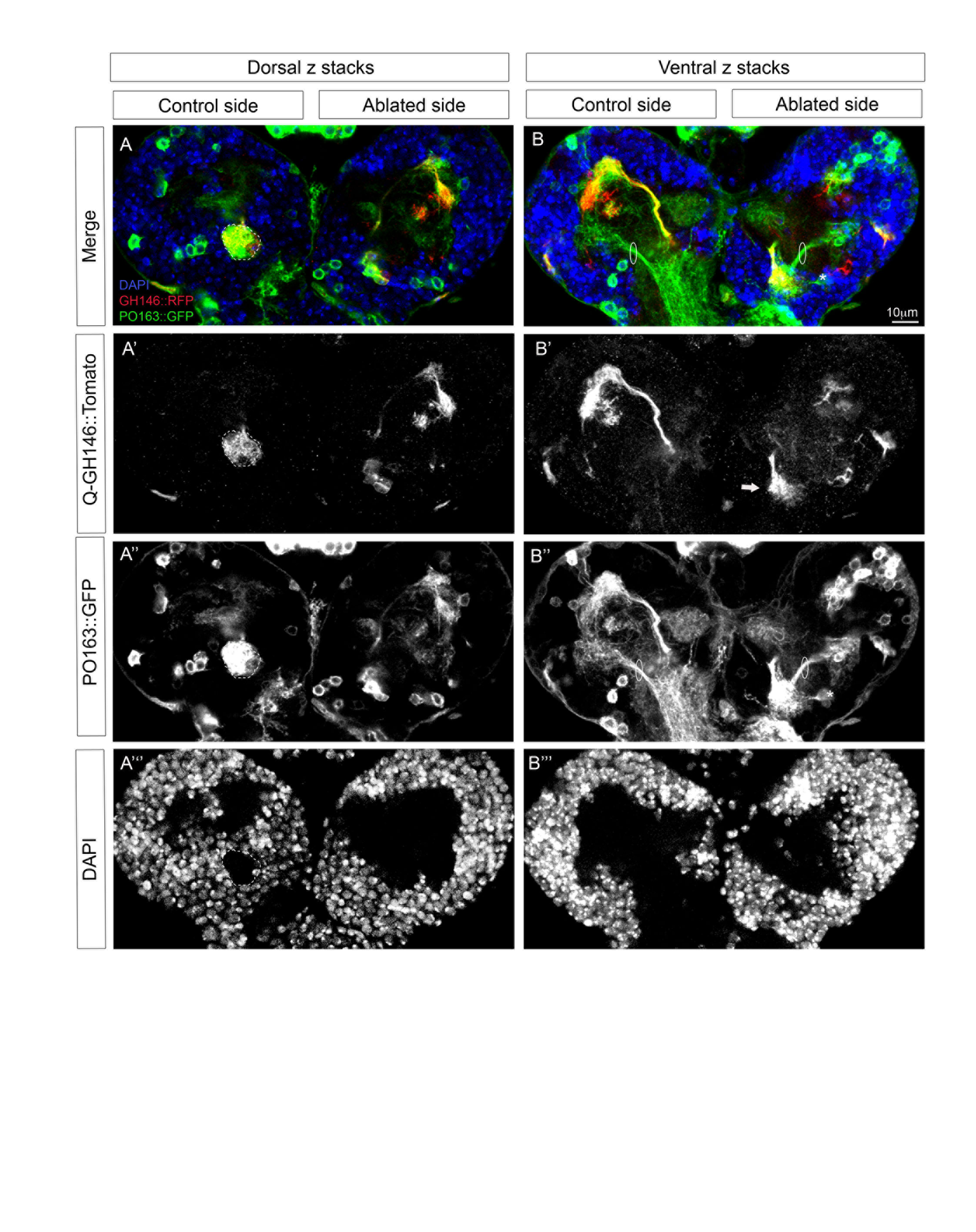

Supplement: Figure S2 — Related to Figure 5. PN dendrites attract ectopic innervation when OSNs are killed early in embryogenesis. (A–A′″) Z projection of dorsal z stacks. The AL is visible on the control side (dashed line), with PN dendrites innervating it, and as a gap in DAPI staining, however on the ablated side, due to a combination of a different mounting orientation of that brain lobe, and to the fact that PN dendrites in the ablated side have grown more ventrally to “find” presynaptic innervation, only the axons and some cell bodies of PNs are visible, but not the dendrites. (B–B′″) Z projection of ventral z stacks. PN dendrites in the OSN ablated side have attracted presynaptic innervation from some nearby cell bodies (asterisk), and an axonal fascicle labelled in the PO163 pattern with GFP that on the control side runs from the brain lobes into the SOG (green fascicle surrounded by a white circle) and that on the ablated side has been “sequestered” by PN dendrites. (TIF) [file pbio.1001400.s002.tif]

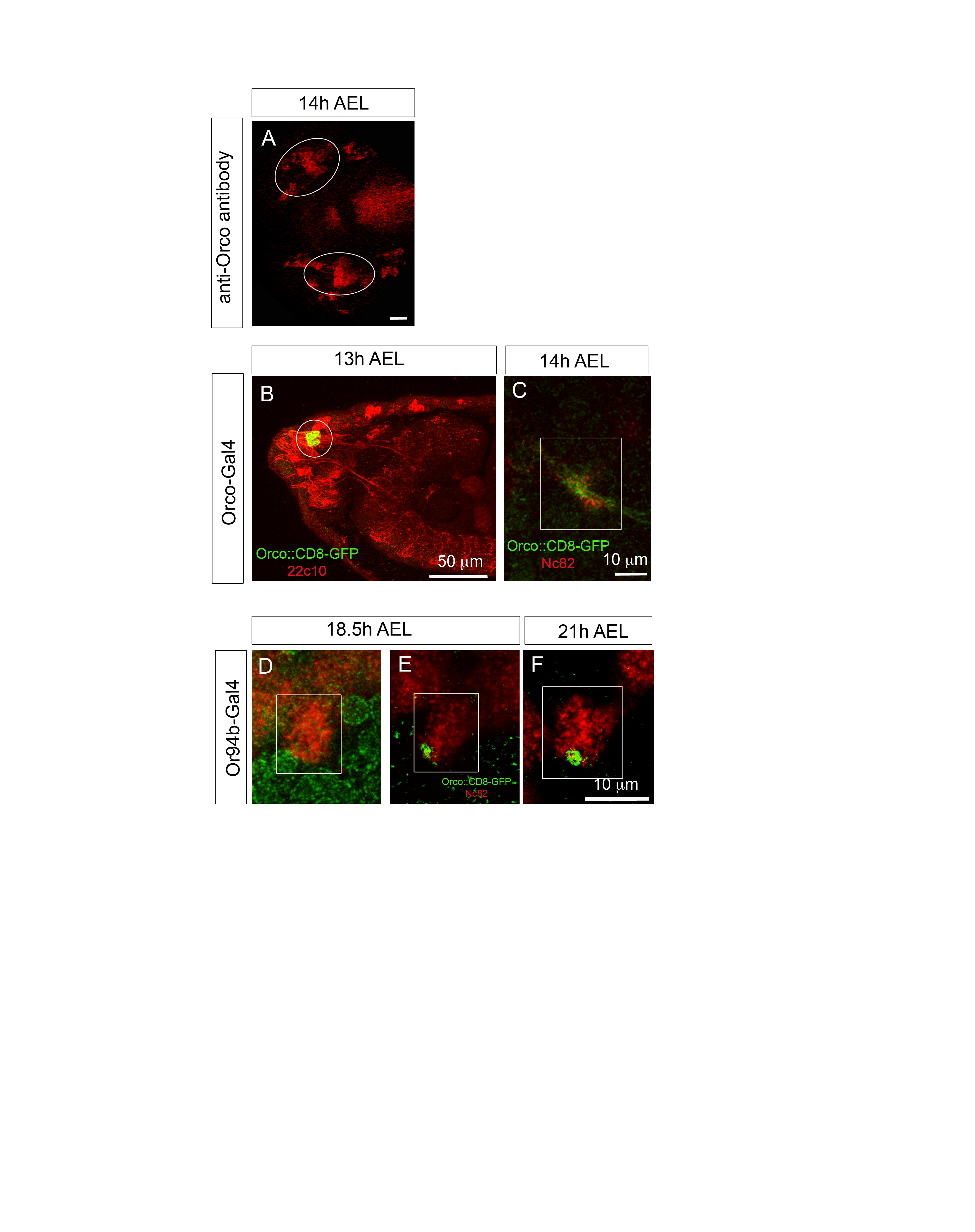

Supplement: Figure S3 — Related to Figure 7. Onset of expression of receptor Gal4 lines. (A) Orco is expressed from early stages; here expression at 14 h AEL is shown with anti-Orco antibody staining. OSNs are surrounded by a white circle. (B–C) Orco Gal4 begins to be expressed at 13 h AEL (B), but GFP cannot be detected in the terminals in the AL until 14 h AEL (C). OSNs are surrounded by a white circle, and AL is enclosed in a white square. (D–F) Or94b-Gal4, a Gal4 for a specific OR, only begins to show expression in the AL at 18.5 h AEL. Half of the screened embryos did not show any expression at 18.5 h AEL (D), but the other half did show a faint expression (E). At hatching time the Gal4 line is reliably expressed in one OSN terminal (F). (TIF) [file pbio.1001400.s003.tif]

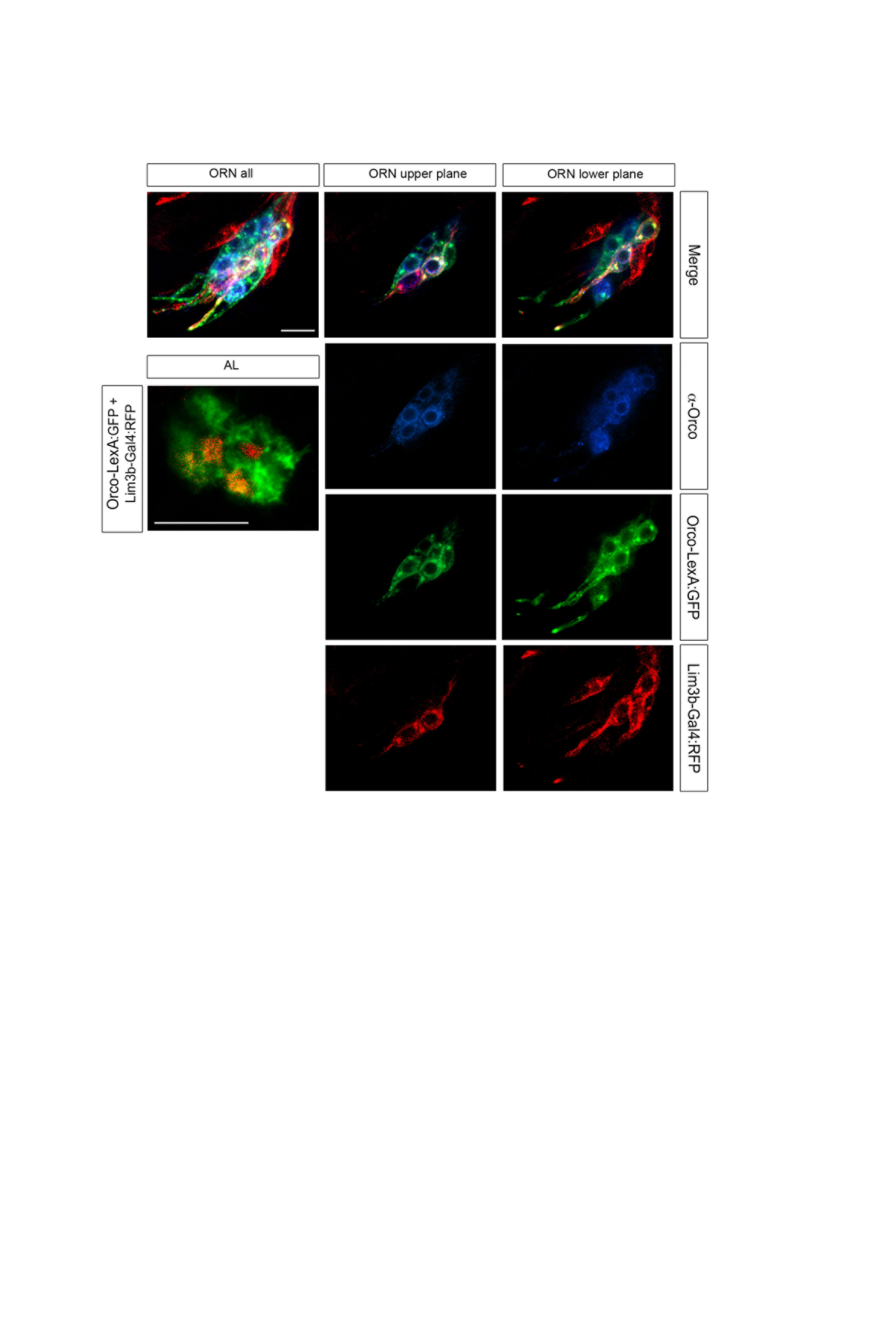

Supplement: Figure S4 — Related to Figure 7. Lim3b Gal4 is expressed in four OSNs. Four cells that express Lim3b Gal4 (red) overlap with anti-Orco antibody staining (blue) that labels all OSNs. Two of the Lim3b positive OSNs have their cell bodies in a dorsal position and send their dendrites together to a common sensillum of the DO. The other two Lim3b positive OSNs are situated in a more ventral position within the DOG and send their dendrites together to another common DO sensillum. The samples are also labelled with Orco-LexA driving mCD8GFP (green), and this line is expressed in all OSNs and marks the contour of the AL, where four glomeruli are labelled by the four Lim3b positive OSNs in red. Scale bar, 5 mm. (TIF) [file pbio.1001400.s004.tif]
